# An analysis on rational use and affordability of medicine after the implementation of National Essential Medicines Policy and Zero Mark-up Policy in Hangzhou, China

**DOI:** 10.1371/journal.pone.0213638

**Published:** 2019-03-14

**Authors:** Wenhui Mao, Yunyu Huang, Wen Chen

**Affiliations:** 1 School of Public Health, Fudan University, Shanghai, China; 2 Duke Global Health Institute, Duke University, Durham, North Carolina, United States of America; University of Campania, ITALY

## Abstract

**Background:**

The National Essential Medicine Policy and the Zero Mark-up Policy was introduced to improve the rational use and affordability of medicine. This study analyzed the changes of medicine use at different Health Care Institutions in Hangzhou city after the implementation of National Essential Medicine Policy and the Zero Mark-up Policy.

**Methods:**

Facility based survey was conducted in 17 Health Care Institutions and 16406 outpatient prescriptions in 2011 and 2013 were collected. Average number of medicines, average number of antibiotics and average expenditure per prescription were analyzed. Comparisons between 2011 and 2013, among different levels of Health Care Institutions and age groups were conducted.

**Results:**

The average number of medicines per prescription, use of antibiotics, intramuscular (IM) injections and intravenous (IV) injections decreased while the use of hormones increased. No significant change of the average medicine expenditure per prescription was observed. Disparities among different levels of Health Care Institutions and different age groups existed.

**Conclusion:**

The problems of poly-pharmacy, overuse of antibiotics, intramuscular (IM) injections and intravenous (IV) injections and hormones still existed, however mitigated after the implementation of The National Essential Medicine Policy and the Zero Mark-up Policy.

## Introduction

China’s health system has made many progress, but the rapidly increasing expenditure on medicines was an issue of concern [1,2]. In 2012, medicine expenditure took up 40.37% of Total Health Expenditure (THE) and the medicine expenditure grew faster than that of gross domestic product (GDP) since 1990 [3]. This growth can be traced back to the market reform in 1980s that public Health Care Institutions (HCIs, refer to all kinds of facilities that provide health care services, including tertiary hospitals, secondary hospital and primary health care centers) could claim at least 15% of the medicine expenditure as revenue to compensate the reduction in government subsidies[4], which was regarded as one of the incentives on health providers to increase revenue by over-prescription [5]. WHO estimated over half of all medicines were prescribed, dispensed or sold inappropriately [6]. A systematic review found that irrational use of medicines in China was severe, featured with polypharmacy, overuse of antibiotics and overuse of injections [7].

To improve the rational use of medicine, the National Essential Medicine Policy (NEMP), along with Zero Mark-up Policy as its matching policy, were introduced in 2009[8]. More specifically, a National Essential Medicine List (NEML) was issued and all of the primary HCIs were required to stock and dispense medicines on the NEML. The Zero Mark-up Policy terminated the economic incentives behind prescriptions by forbidding HCIs from claiming mark-up of medicines and providing subsidies to compensate HCIs’ reduction in revenues.

A number of publications assessed the preliminary impact of the NEMP and Zero Mark-up Policy in pilot cities or counties during 2009–2011 and inconsistent results were reported [10–12]. Chen et al found out the total number of intramuscular (IM) injections and intravenous (IV) injections prescribed for hypertension decreased, especially in pilot sites, while the number of antibiotics and hormones continued to increase. In the meantime, the pilot sites had lower growth rates than control groups. No significant impact on diabetes from NEMP and Zero Mark-up Policy was observed [10]. Yang et al reported that the exclusion of hormones and certain antibiotics from the NEML reduced the (often unwarranted) prescription of hormones and antibiotics [11]

Actions have been taken to further support the implementation of NEMP and Zero Mark-up Policy. First, second edition of NEML with 520 medicines was issued in 2012, which had 213 more medicines than that on the first edition. Secondly, more subsidies were provided to HCIs from the central and provincial government to compensate the reduction in medicine revenue after implementing NEMP and Zero Mark-up Policy. Thirdly, performance-based assessment, which focused on the clinical quality instead of services volume, was used in distributing the income of health professionals to encourage better clinical practice and to eliminate the perverse economic incentive of “more prescription, more bonus”. Fourthly, provincial governments were responsible for price negotiation, biding and centralized procurement of medicines on NEML to enable timely monitor of procurement information and demand from HCIs. In some provinces, the Zero Mark-up Policy was further extended to secondary and tertiary HCIs [9]. For instance, the revenue of essential medicine is required to take at least 40% of the total revenue for secondary HCIs and 25% for tertiary HCIs in Hangzhou since 2013. However, whether these initiatives would improve the rational prescribing and/or reduce the medicine expenditures and the impact of the full implementation of NEMP were not fully assessed yet.

Therefore our study aims to analyze the change of the rational use and affordability of medicine in Hangzhou city, after the implementation of NEMP and Zero Mark-up Policy and their initiatives mentioned in previous paragraph.

## Methods

### Conceptual framework

The 2004 WHO Access to Medicine (ATM) framework was adapted to guide this research. ATM framework has four domains: rational use of medicine, affordable prices, sustainable financing and reliable health and supply systems [13]. This study will provide quantitative evidence on the first two dimensions. Rational use of medicine is defined as patients receiving medications appropriate to their clinical needs, in doses that meet their own individual requirements, for an adequate period of time, and at the lowest cost to them and their community. It requires both rational prescribing from health providers and rational health seeking behavior of patients but providers’ role is more determinant at the point of prescribing and our analysis will take the perspective from provider side.

### Selection of study site and its Policy Background

The research was conducted in Hangzhou city mainly for two reasons. First, Hangzhou was one of the earliest pilots for NEMP and Zero Mark-up Policy that provided relatively long observation time. Second, Hangzhou innovatively scaled up NEMP and Zero Mark-up Policy to secondary and tertiary HCIs that facilitate comparison among different levels of HCIs. Since patients in China are free to use all kinds of HCIs without limitation, assessment on the impact of NEMP and Zero Mark-up Policy in different HCIs will inform future policy decision on whether to scale up NEMP and Zero Mark-up Policy to secondary and tertiary HCIs.

Hangzhou is the capital of Zhejiang Province in Eastern China and GDP per capita is 95,000 Yuan in 2013, above the average of cities in China [14]. The NEMP and Zero Mark-up Policy were fully implemented in primary HCIs of Hangzhou in November, 2010 and all primary HCIs were required to equip with and dispense medicines on NEML and PSEML [15], and mark-up from medicines were no longer allowed at primary HCIs. In 2013, a document released by the Hangzhou Government required that at least 40% of prescribed medicine in secondary HCIs should be medicines on NEML or PSEML and this rate was 25% for tertiary HCIs [16].

Our research collected data of year 2011 and 2013. In 2011, all primary HCIs have implemented NEMP and Zero Mark-up Policy while secondary and tertiary HCIs were not objectives of NEMP or Zero Mark-up Policy. Two years later, the policy settings for primary HCIs remained the same but secondary and tertiary HCIs were required to some extent to implement NEMP and Zero Mark-up Policy.

### Sample size and data collection

All 9 tertiary HCIs in Hangzhou were included. A simple randomized sampling was performed to select 10% of secondary HCIs and 10% of primary HCIs [14]. In all, 6, 2 and 9 HCIs from primary, secondary and tertiary levels were selected accordingly.

Health facility based surveys were conducted to collect general information from HCIs, and prescription surveys in the same HCIs were collected for analysis of prescription pattern (questionnaires can be found in S1–S4 Tables). All survey questionnaires including prescription survey were adapted from the National Essential Medicine Evaluation study in 2011, where pilot study on validity of questionnaire has been performed. A structured questionnaire was completed by the head of financial department of selected HCIs in 2011 and 2013 to collect number of prescriptions, annual revenue, and annual revenue from medicines. Prescription survey was used through a systematic random sampling method in the selected HCIs. 125 outpatient prescriptions on March 12, June 12, September 12 and December 12 were extracted in both 2011 and 2013 (500 prescriptions for each year) with the basic information of patients (gender, age), name, quantity and cost of each medicine and the total cost of prescription. If HCI had less than 125 prescriptions on selected date, prescriptions from the next day were collected. Both medicines and injections on the prescription were included for further analysis. Senior medical students coded the generic name of the medicines into antibiotics, hormones, IV injections and IM injections according to 2010 Chinese Pharmacopoeia.

### Ethics approval and consent to participate

Before any research activities, the research protocol was reviewed and approved by the IRB of Fudan University (IRB Director: jiangqw@fudan.edu.cn). Our study doesn’t include human subjects but we did analysis on the prescription. To protect the privacy of patients, no identifiable information (name, ID number, etc.) was collected and prescriptions were randomly recoded for further analysis. No written informed consent was necessary because no personal identifiable information was collected. The Health and Family Planning Committee of Zhejiang Province is one of our collaborators; we obtained their permission to use the related information for academic purpose.

### Data analysis

According to the WHO manual for drug use indicator [17], average number of medicines per prescription, average number of antibiotics per prescription, average number of IM injections per prescription, average number of IV injections per prescription, and average number of hormones per prescription were calculated as the key indicators of rational use of medicines. More information about the definition of each indicators can be found in WHO manual [17].

Average expenditure per prescription and the share of medicine revenue to total revenue of HCIs were calculated as the key indicators of medicines expenditures.

Indicators were also calculated for primary, secondary and tertiary HCIs.

The sample was divided into four age groups to observe the differences among different age groups: group1: 0~17 years old; group2: 18–44 years old; group 3: 45~60 years old; group 4: over 60 years old.

Statistic tests (α = 0.05) were performed to identify the changes from 2011 and 2013. More specifically, the same indicators from different years were tested.

## Results

### General information

8211 and 8195 prescriptions were collected respectively for 2011 and 2013, and 7 HCIs didn’t have required prescriptions (500) on the selected sample date. 12% of the prescriptions were dispensed for age group 1, 23% to group 2, 25% to group 3 and 40% to group 4. Age groups distributed unevenly in different levels of HCIs. Age group 4 (the elderly) was frequent users of primary HCIs while secondary and tertiary HCIs were more frequently used by children and adults (Table 1).

**Table 1:** Number of prescriptions of different age groups in each level of HCIs.

	2011					2013				
HCIs Level	Age group 1	Age group 2	Age group 3	Age group 4	Subtotal	Age group 1	Age group 2	Age group 3	Age group 4	Subtotal
**Primary**	179	559	1227	2254	4219	156	660	1216	2284	4316
	4.24%	13.25%	29.08%	53.42%	-	3.61%	15.29%	28.17%	52.92%	-
**Secondary**	215	339	193	250	997	239	194	164	159	756
	21.56%	34.00%	19.36%	25.08%	-	31.61%	25.66%	21.69%	21.03%	-
**Tertiary**	529	988	638	696	2851	611	915	588	656	2770
	18.55%	34.65%	22.38%	24.41%	-	22.06%	33.03%	21.23%	23.68%	-
**Total**	923	1886	2058	3200	8067	1006	1769	1968	3099	7842
	11.44%	23.38%	25.51%	39.67%	-	12.83%	22.56%	25.10%	39.52%	-

### Number of medicines per prescription

The average number of medicines per prescription significantly declined from 2.27 in 2011 to 2.19 in 2013 and different levels of HCIs had significantly changed in the same period. Secondary HCIs had the highest average number, followed by tertiary and primary HCIs in 2011. This rank changed to: tertiary > primary > secondary in 2013. Secondary HCIs had the most drop of this indicator during 2011 to 2013, from 2.38 to 1.97. In contrast, the average number of medicines per prescription increased 0.09 in tertiary HCIs (Table 2).

**Table 2:** Prescription pattern: Number of medicines per prescription, use of antibiotics, IM injections, IV injections and hormones.

	2011						2013					
**HCIs Level**	**Average # of medicines per prescription**	**# of medicines per prescription**					**Average # of medicines per prescription**	**# of medicines per prescription**				
		**1**	**2**	**3**	**4**	**≥5**		**1**	**2**	**3**	**4**	**≥5**
**Primary**†	2.24±1.50**	1916	1034	543	372	468	2.13±1.43	2056	1045	583	330	379
		44.22%	23.86%	12.53%	8.59%	10.80%		46.80%	23.79%	13.27%	7.51%	8.63%
**Secondary**††	2.38 ±1.47**	361	278	145	106	110	1.97±1.26	486	273	116	65	60
		36.10%	27.80%	14.50%	10.60%	11.00%		48.60%	27.30%	11.60%	6.50%	6.00%
**Tertiary**†	2.29±1.41**	1116	771	447	256	288	2.38±1.49	1070	706	405	294	327
		38.78%	26.79%	15.53%	8.90%	10.01%		38.19%	25.20%	14.45%	10.49%	11.67%
**Total**†	2.27±1.47*	3393	2083	1135	734	866	2.19±1.44	3612	2024	1104	689	766
		41.32%	25.37%	13.82%	8.94%	10.55%		44.08%	24.70%	13.47%	8.41%	9.35%
	**Average # of antibiotics per prescription**	**# of antibiotics per prescription**					**Average # of antibiotics per prescription**	**# of antibiotics per prescription**				
		**0**	**1**	**2**	**3**	**≥4**		**0**	**1**	**2**	**3**	**≥4**
**Primary**††	0.39±0.73 (Average number of antibiotics of all prescriptions)	3079	972	202	47	33	0.35 ±0.78	3286	856	148	51	50
	1.33±0.76** (Average number of antibiotics of prescriptions with antibiotics)	71.06%	22.43%	4.66%	1.08%	0.76%	1.41±0.95	74.83%	19.49%	3.37%	1.16%	1.14%
**Secondary**††	0.56±0.67**	531	381	81	7	0	0.40±0.57	638	329	32	1	0
	1.20±0.44**	53.10%	38.10%	8.10%	0.70%	0.00%	1.09±0.30	63.80%	32.90%	3.20%	0.10%	0.00%
**Tertiary**†	0.35±0.61**	2054	650	158	15	1	0.32±0.56	2034	658	96	14	0
	1.23±0.47**	71.37%	22.59%	5.49%	0.52%	0.03%	1.16±0.42	72.59%	23.48%	3.43%	0.50%	0.00%
**Total**††	0.40±0.69**	5664	2003	441	69	34	0.35±0.69	5958	1843	276	66	50
	1.28±0.63	68.98%	24.39%	5.37%	0.84%	0.41%	1.27 ±0.74	72.72%	22.49%	3.37%	0.81%	0.61%
	**Average # of IM injections per prescription**	**# of IM injections per prescription**					**Average # of IM injections per prescription**	**# of IM injections per prescription**				
		**0**	**1**	**2**	**3**	**≥4**		**0**	**1**	**2**	**3**	**≥4**
**Primary**†	0.11 ±0.49**	4060	120	109	29	15	0.09±0.42	4138	164	60	14	15
	1.79±0.91	93.70%	2.77%	2.52%	0.67%	0.35%	1.54±0.90	94.24%	3.73%	1.37%	0.32%	0.34%
**Secondary**†	0.45 ±0.88**	737	131	95	24	13	0.34±0.74	776	139	61	18	6
	1.71±0.90	73.70%	13.10%	9.50%	2.40%	1.30%	1.52±0.80	77.60%	13.90%	6.10%	1.80%	0.60%
**Tertiary**†	0.29±0.81**	2448	181	142	68	39	0.35±0.86	2293	219	169	81	40
	1.96±1.07	85.06%	6.29%	4.93%	2.36%	1.36%	1.92±1.03	81.83%	7.82%	6.03%	2.89%	1.43%
**Total**†	0.22±0.68	7245	432	346	121	67	0.21±0.66	7207	522	290	113	61
	1.84±0.98	88.24%	5.26%	4.21%	1.47%	0.82%	1.73 ±0.97	87.97%	6.37%	3.54%	1.38%	0.74%
	**Average # of IV injections per prescription**	**# of IV injections per prescription**					**Average # of IV injections per prescription**	**# of IV injections per prescription**				
		**0**	**1**	**2**	**3**	**≥4**		**0**	**1**	**2**	**3**	**≥4**
**Primary**†	0.20±0.85**	4072	40	48	46	127	0.13±0.68	4194	48	49	27	73
	3.25±1.48	93.98%	0.92%	1.11%	1.06%	2.93%	2.88±1.59	95.51%	1.09%	1.12%	0.61%	1.66%
**Secondary**†	0.75 ±1.50**	750	42	79	26	103	0.53 ±1.23	801	37	80	22	60
	3.00±1.50*	75.00%	4.20%	7.90%	2.60%	10.30%	2.67±1.36	80.10%	3.70%	8.00%	2.20%	6.00%
**Tertiary**†	0.43±1.29**	2519	74	26	61	198	0.56 ± 1.46	2362	77	40	61	262
	3.47±1.64	87.53%	2.57%	0.90%	2.12%	6.88%	3.60±1.63	84.30%	2.75%	1.43%	2.18%	9.35%
**Total**	0.35 ±1.13*	7341	156	153	133	428	0.33 ±1.10	7357	162	169	110	395
	3.27±1.57	89.40%	1.90%	1.86%	1.62%	5.21%	3.21±1.62	89.80%	1.98%	2.06%	1.34%	4.82%
	**Average # of hormones per prescription**	**# of hormones per prescription**					**Average # of hormones per prescription**	**# of hormones per prescription**				
		**0**	**1**	**2**	**3**	**≥4**		**0**	**1**	**2**	**3**	**≥4**
**Primary**	0.05±0.27	4123	195	7	4	4	0.06±0.34	4190	173	14	4	10
	1.13±0.55**	95.15%	4.50%	0.16%	0.09%	0.09%	1.31±0.94	95.42%	3.94%	0.32%	0.09%	0.23%
**Secondary**	0.10±0.32	904	90	6	0	0	0.11±0.32	897	99	4	0	0
	1.06±0.24	90.40%	9.00%	0.60%	0.00%	0.00%	1.04±0.19	89.70%	9.90%	0.40%	0.00%	0.00%
**Tertiary**	0.12±0.36	2552	296	29	1	0	0.12±0.35	2511	260	30	1	0
	1.10±0.30	88.67%	10.28%	1.01%	0.03%	0.00%	1.11±0.32	89.61%	9.28%	1.07%	0.04%	0.00%
**Total**	0.08±0.31	7579	581	42	5	4	0.08±0.34	7598	532	48	5	10
	1.10±0.40**	92.30%	7.08%	0.51%	0.06%	0.05%	1.16±0.61	92.74%	6.49%	0.59%	0.06%	0.12%

Significant difference between the average number of 2011 and 2013,

* for P<0.05,

** for P<0.01 (t test)

Significant difference of the proportions of 2011 and 2013,

^†^ for P<0.05,

^††^ for P<0.01 (Pearson Chi-square test or Fisher exact test)

More than 40% of the prescriptions were dispensed with one medicine and about 25% with two medicines. The proportion of prescriptions with 5 or more medicines, regarded as polypharmacy, decreased from 10.55% to 9.35%. This proportion decreased by 4.5% in secondary HCIs, and 3.29% in primary HCIs. On the contrary, the proportion of prescriptions with 5 or more medicines increased by 1.7% in 2013 in tertiary hospitals (Table 2).

Age group 1 (children) had the highest average number of medicines per prescription, followed by group 2 and 4. From 2011 to 2013, the average number of medicines per prescription significantly decreased for age group1, 3 and 4 while no significant change was observed for age group 2. But this indicator was found to be significantly higher in 2013 than 2011 for age group 2 and 4 in tertiary HCIs (Table 3).

**Table 3:** Prescription patterns of different age groups.

HCIs Level	2011				2013			
	Age 1	Age 2	Age 3	Age 4	Age 1	Age 2	Age 3	Age 4
**Average # of medicines per prescription**								
**Primary**	1.98±1.38	2.19±1.44*	2.28±1.51**	2.30±1.53**	1.85±1.29	2.01±1.35	2.15±1.44	2.19±1.46
**Secondary**	2.74±1.68**	2.41±1.47*	2.21±1.30	2.17±1.34**	2.12±1.33	2.08±1.34	1.77±1.18	1.62±1.07
**Tertiary**	2.47±1.51	2.28±1.42*	2.21±1.29	2.24±1.43*	2.54±1.55	2.37±1.50	2.22±1.39	2.38±1.51
**Total**	2.44±1.55*	2.28±1.44	2.25±1.43*	2.28±1.50*	2.33±1.48	2.21±1.44	2.14±1.41	2.20±1.46
**% of prescriptions with antibiotics**								
**Primary**	51.40%††	47.23%†	29.42%	23.07%†	27.56%	41.43%	28.29%	19.19%
**Secondary**	63.26%††	49.85%	43.01%	31.60%††	45.19%	42.78%	32.93%	13.84%
**Tertiary**	49.15%†	29.35%	21.94%	18.10%	43.21%	29.40%	18.54%	16.92%
**Total**	52.87%††	38.34%	28.38%	22.66%††	41.25%	35.35%	25.76%	18.43%
**Average # of antibiotics per prescription (for those with antibiotics)**								
**Primary**	1.11±0.35**	1.36±0.73	1.33±0.75*	1.37±0.83	1.30±0.71	1.41±0.90	1.45±0.98	1.39±0.99
**Secondary**	1.18±0.43**	1.27±0.51**	1.18±0.39**	1.13±0.34*	1.07±0.26	1.13±0.38	1.07±0.26	1.05±0.21
**Tertiary**	1.17±0.41**	1.27±0.48*	1.25±0.51	1.25±0.52	1.08±0.28	1.21±0.48	1.23±0.46	1.21±0.47
**Total**	1.16±0.40**	1.30±0.59	1.29±0.66*	1.32±0.75	1.10±0.35	1.29±0.70	1.36±0.85	1.34±0.90
**% of prescriptions with IM injections**								
**Primary**	19.55%	11.45%	4.73%	4.13%	12.82%	9.86%	4.19%	3.64%
**Secondary**	42.33%	24.78%	21.24%†	18.00%†	35.56%	21.13%	11.59%	8.18%
**Tertiary**	21.17%	16.30%†	11.76%	10.63%	23.57%	19.78%	15.14%	13.41%
**Total**	25.79%	16.38%	8.45%	6.63%	24.75%	16.23%	8.08%	5.94%
**Average # of IM injections per prescription**								
**Primary**	1.74±0.70**	1.88±0.85	1.83±0.84*	1.87±1.10	1.15±0.37	1.49±0.73	1.86±1.11	1.64±1.03
**Secondary**	1.88±0.90	1.65±0.86	1.61±0.92	1.60±0.91	1.45±0.68	1.73±0.78	1.74±1.33	1.62±0.65
**Tertiary**	1.88±0.71*	2.09±1.17*	1.95±1.10	1.84±1.26	1.90±0.85	2.02±1.11	1.85±0.98	1.83±1.19
**Total**	1.86±0.79*	1.93±1.04	1.83±0.98	1.80±1.12	1.68±0.81	1.86±1.01	1.84±1.06	1.73±1.09
**% of prescriptions with IV injections**								
**Primary**	24.58%	14.49%†	4.24%	3.50%	17.31%	8.65%	3.54%	2.85%
**Secondary**	43.26%	24.19%	19.17%†	14.40%†	35.15%	18.04%	9.76%	5.03%
**Tertiary**	21.55%	13.26%†	8.78%†	7.18%	23.57%	17.27%	12.24%	9.15%
**Total**	27.19%	15.59%	7.05%	5.16%	25.35%	14.14%	6.66%	4.29%
**Average # of IV injections per prescription**								
**Primary**	2.70±1.36	3.07±1.53	3.62±1.32*	3.51±1.49	2.41±1.67	2.79±1.39	3.12±1.71	3.06±1.63
**Secondary**	3.17±1.58*	2.88±1.46	2.78±1.47	3.08±1.46	2.60±1.33	3.40±1.22	2.69±1.78	3.25±1.16
**Tertiary**	3.60±1.29	3.61±1.74	3.16±1.81	3.34±1.87	3.67±1.39	3.61±1.68	3.46±1.73	3.57±1.97
**Total**	3.28±1.45	3.26±1.64	3.23±1.58	3.36±1.61	3.18±1.51	3.39±1.59	3.25±1.73	3.30±1.78
**% of prescriptions with hormones**								
**Primary**	3.91%	6.26%	4.65%	4.79%	5.13%	4.55%	4.52%	4.69%
**Secondary**	11.16%	12.39%	7.25%	6.40%	16.74%	8.76%	6.71%	6.29%
**Tertiary**	14.56%	10.43%	11.76%	10.06%	11.62%	10.82%	9.52%	9.60%
**Total**	11.70%	9.54%	7.09%	6.06%	11.83%	8.26%	6.20%	5.81%
**Average # of hormones per prescription**								
**Primary**	1.00±0.00**	1.03±0.17**	1.16±0.68	1.17±0.57*	1.88±1.13	1.23±0.77	1.29±1.01	1.29±0.93
**Secondary**	1.04±0.20*	1.12±0.33	1.00±0.00	1.00±0.00*	1.00±0.00	1.18±0.39	1.00±0.00	1.10±0.32
**Tertiary**	1.00±0.00**	1.12±0.35	1.16±0.37	1.10±0.30	1.03±0.17	1.15±0.36	1.13±0.38	1.13±0.34
**Total**	1.01±.010**	1.10±0.32**	1.14±0.50	1.13±0.47*	1.08±0.37	1.17±0.48	1.19±0.73	1.22±0.75

Significant difference between the average number of 2011 and 2013,

* for P<0.05,

** for P<0.01 (t test)

Significant difference of the proportions of 2011 and 2013,

^†^ for P<0.05,

^††^ for P<0.01 (Pearson Chi-square test or Fisher exact test).

### Use of antibiotics

The proportion of prescriptions without antibiotics significantly increased from 68.98% to 72.72% from 2011 to 2013, indicating the frequency of antibiotics dispensed decreased. However, the average number of antibiotics per prescriptions did not change significantly. Different levels of HCIs also showed different trends of antibiotics use. For primary HCIs, the average number of antibiotics per prescriptions significantly increased, despite that the proportion of prescriptions without antibiotics increased about 3 percent: the combination use of antibiotics were more frequent in 2013. Secondary and tertiary HCIs, on the other hands, had higher proportions of prescriptions without antibiotics and also lower average numbers of antibiotics per prescriptions in 2013 than those in 2011(Table 2).

Secondary HCIs were more likely to prescribe antibiotics. In 2011, almost half of the prescriptions had at least one antibiotic. This situation was improved in 2013 that less than 40% of the prescriptions contained antibiotics. Less than 30% of prescriptions in tertiary HCIs and primary HCIs contained antibiotics, but the average number of antibiotics dispensed is 1.33 in primary HCIs, followed by 1.23 of tertiary HCIs in 2011. Furthermore, this indicator increased to 1.41 in primary HCIs (Table 2).

Age group 1 was more likely prescribed with antibiotics than other groups and the proportion of prescription with antibiotics dropped significantly from 52.87% in 2011 to 41.25% in 2013. Age group 4 also underwent a slightly decrease from 22.66% to 18.43% (Table 3).

### Use of IM injections

The proportion of prescriptions without IM injections was 88.24% in 2011 and 87.97% in 2013 and no significant change was observed on the average number of IM injection per prescription. The proportion of prescriptions without IM injections increased in primary and secondary HCIs but decreased in tertiary HCIs (Table 2).

Generally, secondary HCIs were most likely to prescribe IM injections, followed by tertiary. Less than 7 percent of the prescriptions in primary HCIs were dispensed with IM injection. The average number of IM injections per prescription was 1.92 for tertiary HCIs in 2013, whereas averagely 1.52 and 1.54 IM injections were prescribed in secondary and primary HCIs in the same year (Table 2).

There was no significant change in proportion of prescriptions with IM injections among different age groups, among which age group 1 had the highest proportion of 25.79% in 2011 and 24.75% in 2013. Only about 6~8% of prescriptions were dispensed with IM injections for age group 3 and 4 (Table 3).

### Use of IV injections

The pattern and trend of IV injection usage was similar to that of IM injections in many perspectives. For instance, the proportion of prescriptions without IV injections in different levels of HCIs was similar to that of IM injections whereas the average number of IV injection per prescription was much higher (3.27 in 2011 for IV injections comparing to 1.84 for IM injections in the same year). (Tables 2 & 3).

### Use of hormones

The proportion of prescriptions without hormones didn’t change significantly but the average number of hormones per prescription increased in primary HCIs. Over 10% of the prescriptions in tertiary HCIs were prescribed with hormones, higher than that in secondary HCIs (about 10%) or primary HCIs (5%) (Table 2).

About 11% of the prescriptions for age group 1 contained hormones, followed by 9.54%, 7.09% and 6.05 for group 2–4 in 2011 (Table 2).

### Average medicines expenditure per prescription

No significant change of the average medicine expenditure per prescription was found between 2011 and 2013. Tertiary HCIs had the highest level of average medicine expenditure per prescription: 64.59 Yuan in 2011 and 61.89 Yuan in 2013. The variation of average medicine expenditure per prescription was also highest in tertiary HCIs. This indicator increased, but not statistically significant, for primary and secondary HCIs from about 38 Yuan to over 40 Yuan (Table 4).

**Table 4:** Average medicine expenditure per prescription and proportion of medicine revenue at share to total revenue of HCIs.

HCIs Level	2011				2013			
	**Average medicine expenditure per prescription** **(RMB)**		**Proportion of medicine revenue as share to total revenue of HCIs**		**Average medicine expenditure per prescription (RMB)**		**Proportion of medicine revenue as share to total revenue of HCIs**	
**Primary**	38.21±54.58		61.75%		40.74±53.13		54.29%	
**Secondary**	39.61±48.54		55.69%		44.96±71.65		58.04%	
**Tertiary**	64.59±131.14		50.26%		61.89±147.02		50.02%	
**Total**	49.49±95.52				49.85±104.47			
**Average medicine expenditure per prescription (RMB)**								
	**Age 1**	**Age 2**	**Age 3**	**Age 4**	**Age 1**	**Age 2**	**Age 3**	**Age 4**
**Primary**	19.70±26.43	32.49±47.44	36.40±47.17*	40.53±57.17	24.40±29.40	29.58±37.30	39.12±48.42	43.56±51.34
**Secondary**	18.56±23.02	40.26±49.52	43.72±55.68	48.05±50.00*	20.18±20.12	40.23±48.06	41.91±48.52	77.53±112.64
**Tertiary**	31.42±75.66	72.26±155.01	61.58±100.13	83.48±148.79	29.59±82.48	61.94±151.55	67.83±119.11	88.73±198.76
**Total**	27.78±27.39	57.56±125.98*	46.02±72.58*	52.66±93.38	27.39±70.49	49.18±119.07	49.41±81.76	56.76±114.20

1 RMB = 0.1548 USD in 2011; 1 RMB = 0.1615 USD in 2013.

*Significant difference between the average number of 2011 and 2013, P<0.05(t test).

Age group 1 remained the lowest average medicine expenditure per prescription (27 Yuan) among all the population. This indicator was 57.56 Yuan for group 2 in 2011 and reduced to 49.18 Yuan in 2013. The average medicine expenditure per prescription of age group 3 and 4 increased slightly, from 46.02 Yuan to 49.41 Yuan and 52.66 Yuan to 56.76 Yuan (Table 4).

### Share of medicine revenue as to total revenue

Share of medicine revenue as to total revenue of HCIs was over 50%, indicating that medicine is the main source of revenue for all HCIs. This indicator was 61.75% at primary HCIs, followed by 55.69% of Secondary and 50.26% of Tertiary HCIs in 2011. In 2013, share of medicine revenue as to total revenue of HCIs dropped from 61.75% to 54.29%, while tertiary HCIs remained at the same level, and secondary HCIs witness a slight increase from 55.69% to 58.04% (Table 4).

## Discussion

After the full implementation of NEMP and Zero Mark-up Policy, Hangzhou city made moderate progress in in the rational prescription of medicines. Average number of medicines per prescription, average number of antibiotics per prescription and average number of IV injections per prescription were significantly reduced. Although no significant change was observed on the average number of IM injections, the proportion of prescription with IM injections decreased. Hormones, on the contrary, were witnessed a slight rise. In brief, the overuse of antibiotics, IV injections and IM injections were reduced and more efforts should be invested to reduce the overuse of hormones.

According to a systematic review on rational use of medicine in China [7], the median of number of medicines per prescription was 2.94, much higher than that in Hangzhou (2.27 for 2011 and 2.19 for 2013). The median of proportion of prescriptions with antibiotics was 52.6%, over 20% higher than that in Hangzhou. The median of proportion of prescriptions with IM injections from the review was triple of that in Hangzhou. Attributed to the Hangzhou’s great investment on health services delivery system, Hangzhou achieved better performance on rational use of medicines than many other cities in China. Its experience can be shared with cities with similar economic development level and health services provision systems. Residents in Hangzhou shared 7.37 hospital beds and 11.09 health professionals per 1000 population in 2013, among which 4.20 were registered doctors and 4.39 were nurses. Many primary HCIs were built within 15 minutes’ walk for nearby residents [14]. The easy access to health services promoted the rational use of medicines by reducing self-medications and patients did not need to take excessive medicines per outpatient visit.

However, the improvement on rational use of medicines still has a long way to go. First, average number of medicines per prescription was much higher than the recommended level by WHO [18]. Secondly, abuse or overuse of antibiotics may lead to resistance and even severe adverse drug reaction. Herein, the use of antibiotics, the most commonly abuse or overuse and expensive medicine that still took up to 30% of all prescriptions, should be monitored and regulated. Thirdly, the abuse and overuse of IM injection and IV injections is a tremendous problem in China and requires more attention. Fourthly, hormones were more frequently used in 2013 than in 2011 while its necessity was still questionable.

Economic incentive and profits from prescribing medicines was the most frequently mentioned influential factor for irrational use of medicine in China [7]. The implementation of Zero Mark-up Policy in primary HCIs since 2010 might, to some extent, account for the observed improvement on the rational use of medicines in primary HCIs. However, secondary or tertiary HCIs haven’t made convincing progress. One possible explanation is that NEMP was not fully implemented in secondary and tertiary HCIs. Considering the high service volumes in secondary and tertiary HCIs as well as the complexity of cases, any policy designed for secondary and tertiary HCIs will call for more precaution. It is time to return to the nature of medicine, which is to provide treatment for diseases, and clinical demand should be regarded as the core of rational use of medicines. We will need more emphasis on clinical guideline, along with matching policies such as strict regulations on over-prescription, and economic incentives for a better clinical quality.

Additionally, different levels of HCIs have different performances and achievements on the rational use of medicine. Primary HCIs need more attentions on the combination use of antibiotics. On the other hand, secondary HCIs, which had almost 40% of the prescriptions with antibiotics, are required to better comply with the clinical guideline for the indications of antibiotics. Both secondary and tertiary HCIs may need to enhance the control over IV injections and hormones.

The four age groups had different preference of the utilization of the levels of HCIs. The elderly was more often to use primary HCIs whiles secondary and tertiary HCIs were more frequently used by children and adults. The prescription pattern also showed variety among age groups. In brief, children and the elderly (age group 1 and 4) were prescribed with more medicines, antibiotics, IM injections and hormones. In the meantime, the intensity of antibiotics, IM injections and hormones use were higher for the elderly. Both children and the elderly were found with more significant changes on these indicators, while prescription patterns for age group 2 and 3 did not change much during 2011 to 2013. One possible explanation may be that children (especially infants and children under 5 years old) and the elderly have more health service need, which also yielded more potentials for changes. Further study should focus on the reactions of different age groups to policy changes.

Among all the indicators, no significant change of the average medicine expenditure per prescription was found between 2011 and 2013. Many factors were closely related to the medicine expenditures, such as medicine category, number of medicines per prescription, the unit price for each medicine and the total volume of each medicine. Even though the Zero Mark-up Policy disconnected the economic incentives for dispensing expensive medicines or over-dispensing, the knowledge from both patients and doctors did not improve at the same pace. Many reports indicated that patients might require more medicines for family storage or even for relatives [9]. What’s more, many patients held a belief that expensive medicines must have better curative effect than the inexpensive ones [7]. The reimbursement from health insurance also reduced patients’ sensitivity on total expenditure [19]. More supportive policies and public education campaigns should be developed to enhance the awareness of rational use of medicines.

Irrational use of medicine, especially poly-pharmacy and over-use of antibiotics, has been observed globally for different causes [20–23]. It requires interventions from both provider and patient side. China’s experience of NEMP with a list of essential medicine could be considered as polity options to improve the rational use of medicine, especially for primary health level.

Lack of control group(s) is a major limitation of our study. All of the primary HCIs implemented the NEMP and Zero-Markup Policy in similar time and left no blank control groups behind. However, primary HCIs were required to use 100% medicines on NEML only while 40% and 25% of total medicines prescribed in secondary and tertiary HCIs were expected to be medicines on NEML. Based on this feature, we assumed that different levels of HCIs would have different reactions towards NEMP and Zero-Markup Policy and we conducted inner-groups comparison on different levels of HCIs to observe the changes in prescribing behavior after the implementation of NEMP and Zero-Markup Policy. Similar to other cross-sectional health policy research, our evidence is not strong enough to make causal inferences between the implementation of NEMP and Zero-Markup Policy and the improvements on rational use of medicine and the results should be interpreted with caution.

## Conclusion

The rational use of medicines was improved while the impact on affordability of medicines was not revealed. However, the problem of polypharmacy, overuse of antibiotics, IM injections, IV injections and hormones still existed. The prescription pattern among different levels of HCIs indicated that primary and secondary HCIs might need further attention and intervention on the use of antibiotics. More actions should be adopted to control the use of IV injections and hormones in secondary and tertiary HCIs.

## Supporting information

S1 Table Prescription survey. (DOCX)

S2 Table Prescription survey in original language (Chinese). (DOCX)

S3 Table Health facility based survey. (DOCX)

S4 Table Health facility based survey in original language (Chinese). (DOCX)

S5 Table Data. (XLSX)

## Abbreviations

HCIs: Health Care Institutions
NEMP: National Essential Medicine Policy
NEML: National Essential Medicine List
PSEML: Provincial Supplementary Essential Medicine List
IM injection: intramuscular injections
IV injection: intravenous injections
