# Supplementary material for: An analysis on rational use and affordability of medicine after the implementation of National Essential Medicines Policy and Zero Mark-up Policy in Hangzhou, China
Source: PLoS One. 2019 Mar 14;14(3):e0213638. doi: 10.1371/journal.pone.0213638 (PMC6417690; doi:10.1371/journal.pone.0213638)
Supplement: S1 Table — (DOCX) [file pone.0213638.s001.docx]

**S1 Table. Prescription Survey**

| Hos-pital  ID | Pres  crip  tion  ID | Age | Gen  der | Name  of  the med-icine | Chinese medicine  ①Yes  ②No | Western  Medicine  ①Yes  ②No | Anti-biotics  ①Yes  ②No | IM  Inject-ions  ①Yes  ②No | IV  Inject-ions  ①Yes  ②No | Hor-mone  ①Yes  ②No | Genetic  Name  ①Yes  ②No | On National/  Provincial  Essential  Medicine List  ①Yes  ②No | On  the  reimbur-  sable  list  of  insurance  ①Yes  ②No | Qua-  ntity | Unit Price | Total cost  of  the medi-cine | Total  cost  of the prescri-ption |
| --- | --- | --- | --- | --- | --- | --- | --- | --- | --- | --- | --- | --- | --- | --- | --- | --- | --- |
|  |  |  |  |  |  |  |  |  |  |  |  |  |  |  |  |  |  |
|  |  |  |  |  |  |  |  |  |  |  |  |  |  |  |  |  |  |
|  |  |  |  |  |  |  |  |  |  |  |  |  |  |  |  |  |  |
|  |  |  |  |  |  |  |  |  |  |  |  |  |  |  |  |  |  |
