# Supplementary material for: An analysis on rational use and affordability of medicine after the implementation of National Essential Medicines Policy and Zero Mark-up Policy in Hangzhou, China
Source: PLoS One. 2019 Mar 14;14(3):e0213638. doi: 10.1371/journal.pone.0213638 (PMC6417690; doi:10.1371/journal.pone.0213638)
Supplement: S2 Table — (DOCX) [file pone.0213638.s002.docx]

**S2 Table. Prescription Survey in original language (Chinese)**

| 医院代码 | 处方编码 | 年龄 | 性别 | 药品名称 | 属于中成药品①是②否 | 属于西药①是②否 | 属于抗菌药品①是②否 | 属于针剂品①是②否 | 属于输液①是②否 | 属于激素①是②否 | 采用通用名①是②否 | 属于国家和省级基本药品目录①是②否 | 属于医保/新农合目录①是②否 | 药品数量 | 药品单价 | 药品总金额 | 处方总金额 |
| --- | --- | --- | --- | --- | --- | --- | --- | --- | --- | --- | --- | --- | --- | --- | --- | --- | --- |
|  |  |  |  |  |  |  |  |  |  |  |  |  |  |  |  |  |  |
|  |  |  |  |  |  |  |  |  |  |  |  |  |  |  |  |  |  |
|  |  |  |  |  |  |  |  |  |  |  |  |  |  |  |  |  |  |
|  |  |  |  |  |  |  |  |  |  |  |  |  |  |  |  |  |  |
