# Supplementary material for: An analysis on rational use and affordability of medicine after the implementation of National Essential Medicines Policy and Zero Mark-up Policy in Hangzhou, China
Source: PLoS One. 2019 Mar 14;14(3):e0213638. doi: 10.1371/journal.pone.0213638 (PMC6417690; doi:10.1371/journal.pone.0213638)
Supplement: S3 Table — (DOCX) [file pone.0213638.s003.docx]

**S3 Table. Health facility based survey**

Name of the facility:_____________________________

Level of the facility:_____________________________

Annual total revenue: 2013_____________________; 2011_______________________

Annual total revenue from medicine: 2013_____________________; 2011_______________________
