# Supplementary material for: An analysis on rational use and affordability of medicine after the implementation of National Essential Medicines Policy and Zero Mark-up Policy in Hangzhou, China
Source: PLoS One. 2019 Mar 14;14(3):e0213638. doi: 10.1371/journal.pone.0213638 (PMC6417690; doi:10.1371/journal.pone.0213638)
Supplement: S4 Table — (DOCX) [file pone.0213638.s004.docx]

**S4 Table. Health facility based survey in original language (Chinese)**

机构名称：_______________________

机构级别：_______________________

本机构年度医疗收入：2013年_________________; 2011年_________________________

本机构年度药品收入：2013年_________________; 2011年_________________________
